# Case of Death from Glanders

**Published:** 1844-04-06

**Authors:** 


#### CASE OF DEATH FROM GLANDERS.

A wealthy wine-grower at Verzy, in the Aube, named Beuzard, having a glandered horse, was, a few days ago, giving him a drench recommended by the veterinary surgeon, when the gag by which the animal's mouth had been kept open, slipped out, and M. Beuzard received a wound in his cheek, from one of the teeth of the horse's upper jaw. The next day his face became dreadfully inflamed, his nostrils ejected a purulent matter, similar to that discharged by the glandered horse, his body was covered with gangrenous spots, and every other symptom of the glanders appeared, and increased in violence until his death, which rapidly ensued.—*Galignani's Messenger.*

At the Hospital of St. Louis, Paris, M. Emery, one of the physicians, treats most of his cases of psoriasis and lepra with an ointment composed of tar, one part; lard, three parts. This treatment is generally successful, but does not prevent the disease returning.

